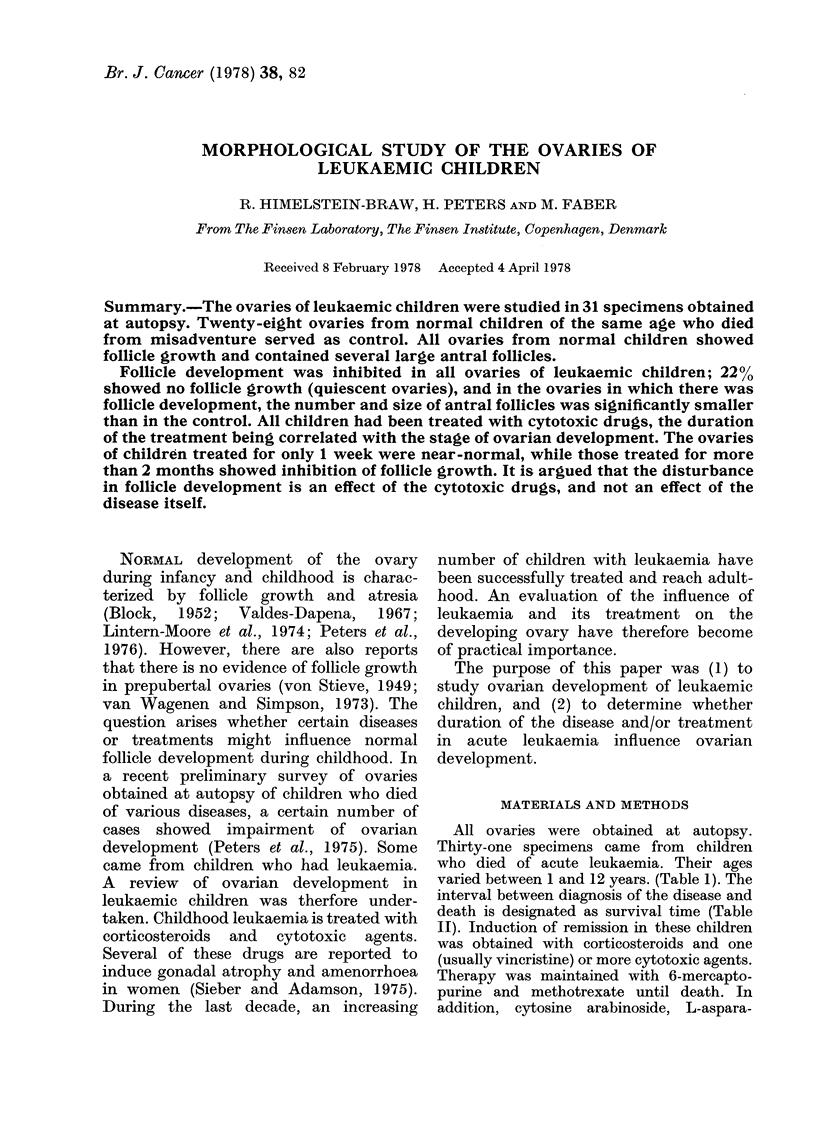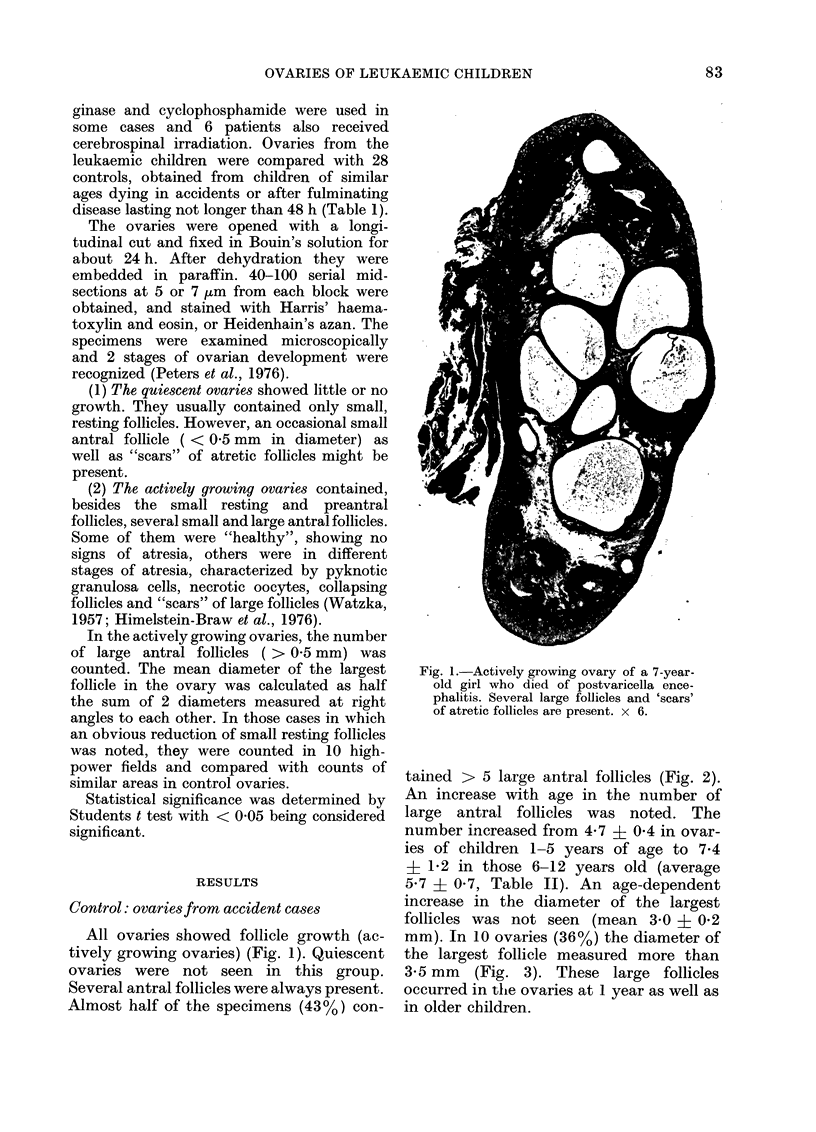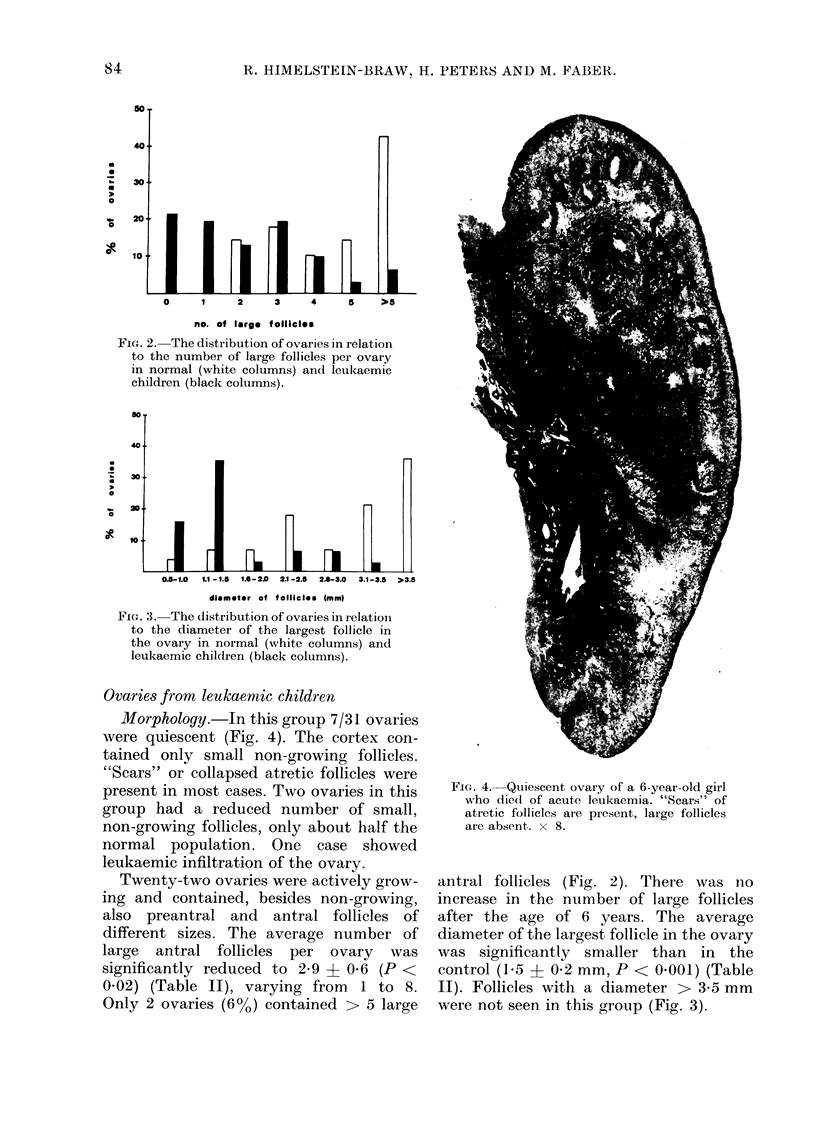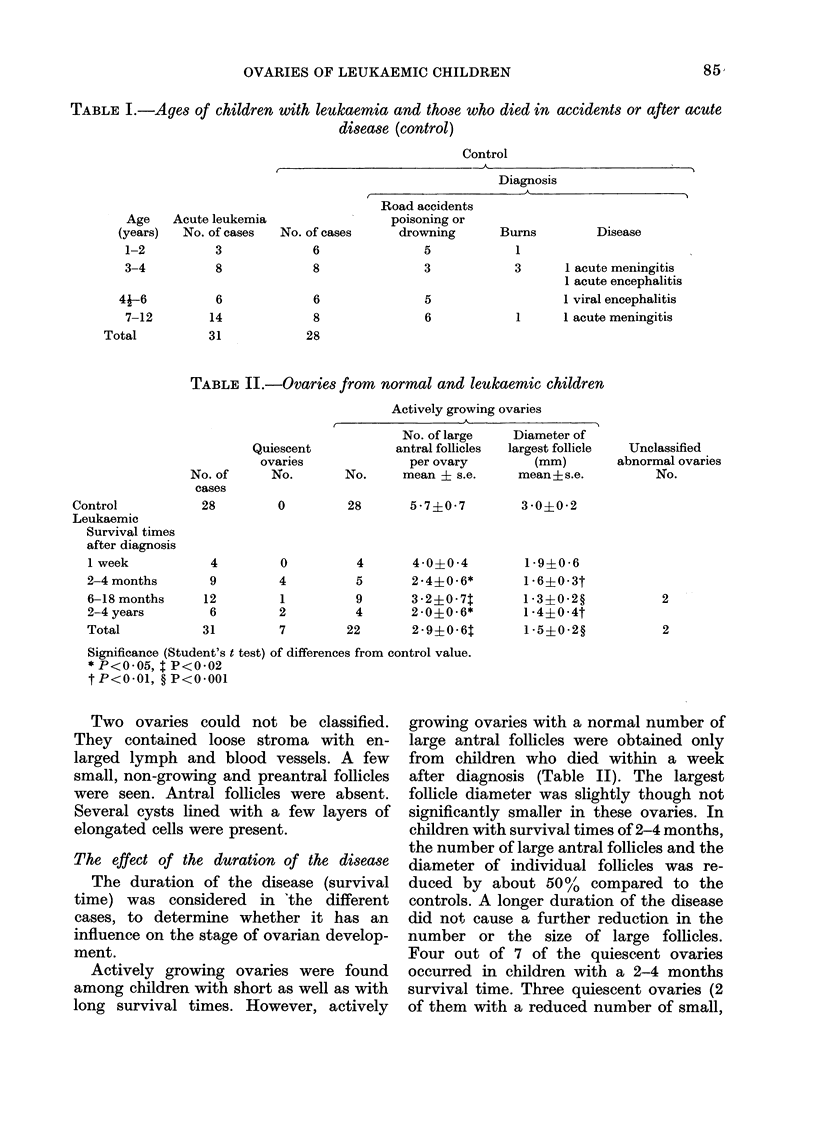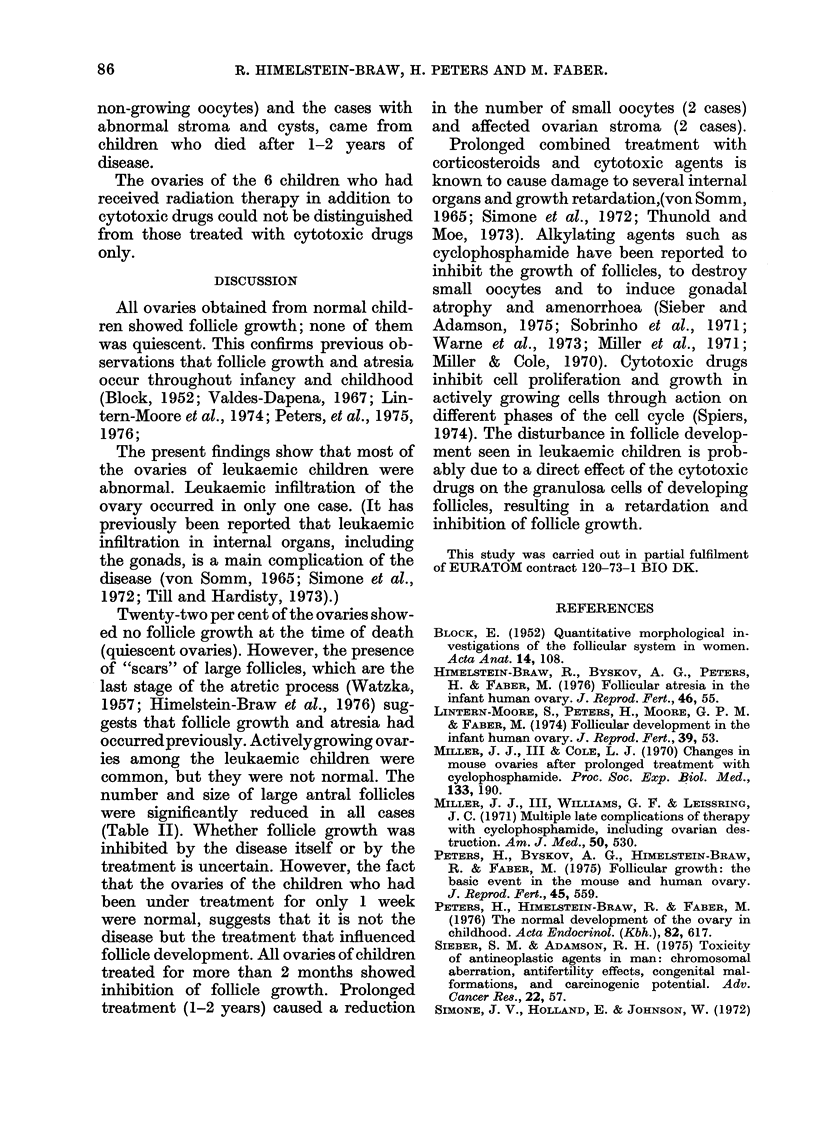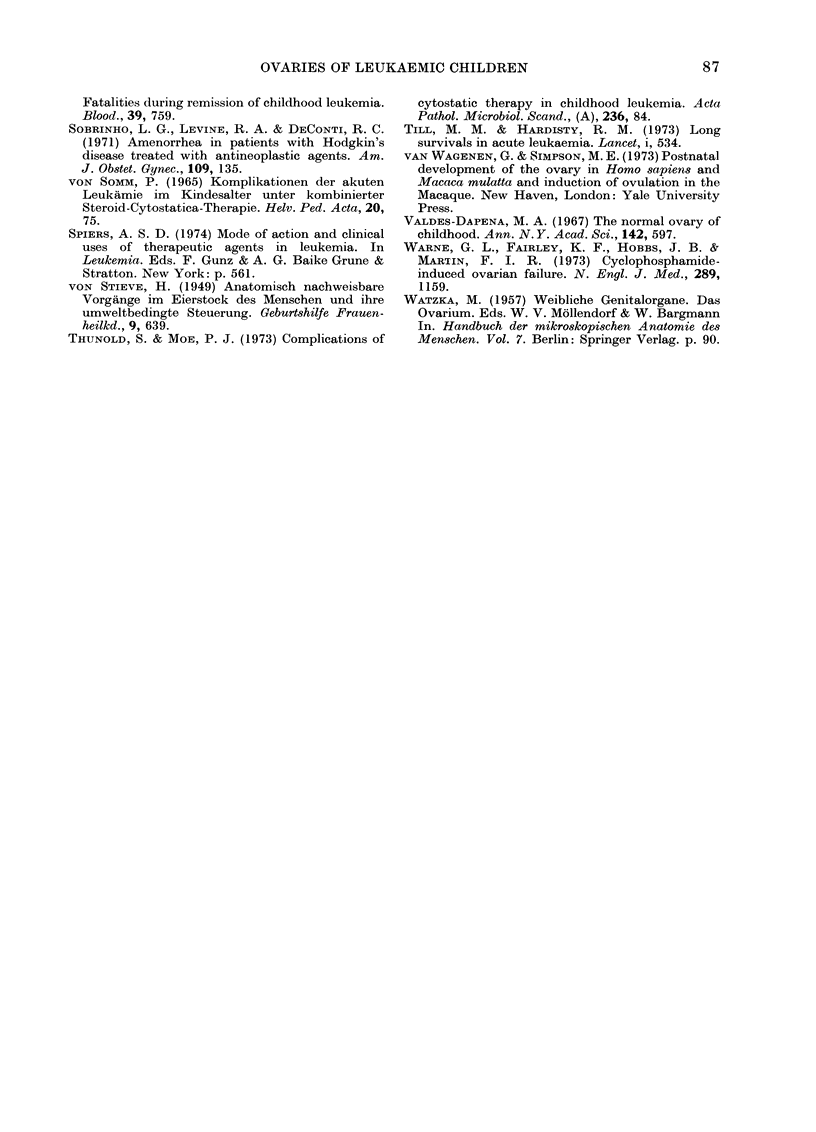# Morphological study of the ovaries of leukaemic children.

**DOI:** 10.1038/bjc.1978.166

**Published:** 1978-07

**Authors:** R. Himelstein-Braw, H. Peters, M. Faber

## Abstract

**Images:**


*Br. J. Cancer* (1978) **38**, 82

### MORPHOLOGICAL STUDY OF THE OVARIES OF LEUKAEMIC CHILDREN

R. HIMELSTEIN-BRAW, H. PETERS AND M. FABER

*From The Finsen Laboratory, The Finsen Institute, Copenhagen, Denmark*

Received 8 February 1978 Accepted 4 April 1978

**Summary.—The ovaries of leukaemic children were studied in 31 specimens obtained at autopsy. Twenty-eight ovaries from normal children of the same age who died from misadventure served as control. All ovaries from normal children showed follicle growth and contained several large antral follicles.**

**Follicle development was inhibited in all ovaries of leukaemic children; 22% showed no follicle growth (quiescent ovaries), and in the ovaries in which there was follicle development, the number and size of antral follicles was significantly smaller than in the control. All children had been treated with cytotoxic drugs, the duration of the treatment being correlated with the stage of ovarian development. The ovaries of children treated for only 1 week were near-normal, while those treated for more than 2 months showed inhibition of follicle growth. It is argued that the disturbance in follicle development is an effect of the cytotoxic drugs, and not an effect of the disease itself.**

Normal development of the ovary during infancy and childhood is characterized by follicle growth and atresia (Block, 1952; Valdes-Dapena, 1967; Lintern-Moore *et al.*, 1974; Peters *et al.*, 1976). However, there are also reports that there is no evidence of follicle growth in prepubertal ovaries (von Stieve, 1949; van Wagenen and Simpson, 1973). The question arises whether certain diseases or treatments might influence normal follicle development during childhood. In a recent preliminary survey of ovaries obtained at autopsy of children who died of various diseases, a certain number of cases showed impairment of ovarian development (Peters *et al.*, 1975). Some came from children who had leukaemia. A review of ovarian development in leukaemic children was therfore undertaken. Childhood leukaemia is treated with corticosteroids and cytotoxic agents. Several of these drugs are reported to induce gonadal atrophy and amenorrhoea in women (Sieber and Adamson, 1975). During the last decade, an increasing number of children with leukaemia have been successfully treated and reach adulthood. An evaluation of the influence of leukaemia and its treatment on the developing ovary have therefore become of practical importance.

The purpose of this paper was (1) to study ovarian development of leukaemic children, and (2) to determine whether duration of the disease and/or treatment in acute leukaemia influence ovarian development.

### MATERIALS AND METHODS

All ovaries were obtained at autopsy. Thirty-one specimens came from children who died of acute leukaemia. Their ages varied between 1 and 12 years. (Table 1). The interval between diagnosis of the disease and death is designated as survival time (Table II). Induction of remission in these children was obtained with corticosteroids and one (usually vincristine) or more cytotoxic agents. Therapy was maintained with 6-mercaptopurine and methotrexate until death. In addition, cytosine arabinoside, L-asparaginase and cyclophosphamide were used in some cases and 6 patients also received cerebrospinal irradiation. Ovaries from the leukaemic children were compared with 28 controls, obtained from children of similar ages dying in accidents or after fulminating disease lasting not longer than 48 h (Table 1).

The ovaries were opened with a longitudinal cut and fixed in Bouin's solution for about 24 h. After dehydration they were embedded in paraffin. 40–100 serial mid-sections at 5 or 7 $\mu$m from each block were obtained, and stained with Harris' haematoxylin and eosin, or Heidenhain's azan. The specimens were examined microscopically and 2 stages of ovarian development were recognized (Peters *et al.*, 1976).

(1) *The quiescent ovaries* showed little or no growth. They usually contained only small, resting follicles. However, an occasional small antral follicle ( $<$ 0·5 mm in diameter) as well as "scars" of atretic follicles might be present.

(2) *The actively growing ovaries* contained, besides the small resting and preantral follicles, several small and large antral follicles. Some of them were "healthy", showing no signs of atresia, others were in different stages of atresia, characterized by pyknotic granulosa cells, necrotic oocytes, collapsing follicles and "scars" of large follicles (Watzka, 1957; Himelstein-Braw *et al.*, 1976).

In the actively growing ovaries, the number of large antral follicles ( $>$ 0·5 mm) was counted. The mean diameter of the largest follicle in the ovary was calculated as half the sum of 2 diameters measured at right angles to each other. In those cases in which an obvious reduction of small resting follicles was noted, they were counted in 10 high-power fields and compared with counts of similar areas in control ovaries.

Statistical significance was determined by Students $t$ test with $<$ 0·05 being considered significant.

Fig. 1.—Actively growing ovary of a 7-year-old girl who died of postvaricella encephalitis. Several large follicles and 'scars' of atretic follicles are present. $\times$ 6.

### RESULTS

### *Control: ovaries from accident cases*

All ovaries showed follicle growth (actively growing ovaries) (Fig. 1). Quiescent ovaries were not seen in this group. Several antral follicles were always present. Almost half of the specimens (43%) contained $>$ 5 large antral follicles (Fig. 2). An increase with age in the number of large antral follicles was noted. The number increased from 4·7 $\pm$ 0·4 in ovaries of children 1–5 years of age to 7·4 $\pm$ 1·2 in those 6–12 years old (average 5·7 $\pm$ 0·7, Table II). An age-dependent increase in the diameter of the largest follicles was not seen (mean 3·0 $\pm$ 0·2 mm). In 10 ovaries (36%) the diameter of the largest follicle measured more than 3·5 mm (Fig. 3). These large follicles occurred in the ovaries at 1 year as well as in older children.

FIG. 2.—The distribution of ovaries in relation to the number of large follicles per ovary in normal (white columns) and leukaemic children (black columns).

FIG. 3.—The distribution of ovaries in relation to the diameter of the largest follicle in the ovary in normal (white columns) and leukaemic children (black columns).

FIG. 4.—Quiescent ovary of a 6-year-old girl who died of acute leukaemia. "Scars" of atretic follicles are present, large follicles are absent. × 8.

*Ovaries from leukaemic children*

*Morphology*.—In this group 7/31 ovaries were quiescent (Fig. 4). The cortex contained only small non-growing follicles. "Scars" or collapsed atretic follicles were present in most cases. Two ovaries in this group had a reduced number of small, non-growing follicles, only about half the normal population. One case showed leukaemic infiltration of the ovary.

Twenty-two ovaries were actively growing and contained, besides non-growing, also preantral and antral follicles of different sizes. The average number of large antral follicles per ovary was significantly reduced to $2{\cdot}9 \pm 0{\cdot}6$ ($P <$ $0{\cdot}02$) (Table II), varying from 1 to 8. Only 2 ovaries (6%) contained $> 5$ large antral follicles (Fig. 2). There was no increase in the number of large follicles after the age of 6 years. The average diameter of the largest follicle in the ovary was significantly smaller than in the control ($1{\cdot}5 \pm 0{\cdot}2$ mm, $P < 0{\cdot}001$) (Table II). Follicles with a diameter $> 3{\cdot}5$ mm were not seen in this group (Fig. 3).

TABLE I.—*Ages of children with leukaemia and those who died in accidents or after acute disease (control)*

| | | Control | | | |
|---|---|---|---|---|---|
| | | | Diagnosis | | |
| Age (years) | Acute leukemia No. of cases | No. of cases | Road accidents poisoning or drowning | Burns | Disease |
| 1–2 | 3 | 6 | 5 | 1 | |
| 3–4 | 8 | 8 | 3 | 3 | 1 acute meningitis<br>1 acute encephalitis |
| 4½–6 | 6 | 6 | 5 | | 1 viral encephalitis |
| 7–12 | 14 | 8 | 6 | 1 | 1 acute meningitis |
| Total | 31 | 28 | | | |

TABLE II.—*Ovaries from normal and leukaemic children*

| | | | Actively growing ovaries | | | |
|---|---|---|---|---|---|---|
| | No. of cases | Quiescent ovaries No. | No. | No. of large antral follicles per ovary mean ± s.e. | Diameter of largest follicle (mm) mean±s.e. | Unclassified abnormal ovaries No. |
| Control | 28 | 0 | 28 | 5·7±0·7 | 3·0±0·2 | |
| Leukaemic | | | | | | |
| Survival times after diagnosis | | | | | | |
| 1 week | 4 | 0 | 4 | 4·0±0·4 | 1·9±0·6 | |
| 2–4 months | 9 | 4 | 5 | 2·4±0·6* | 1·6±0·3† | |
| 6–18 months | 12 | 1 | 9 | 3·2±0·7‡ | 1·3±0·2§ | 2 |
| 2–4 years | 6 | 2 | 4 | 2·0±0·6* | 1·4±0·4† | |
| Total | 31 | 7 | 22 | 2·9±0·6‡ | 1·5±0·2§ | 2 |

Significance (Student's *t* test) of differences from control value.
* $P<0\cdot05$, ‡ $P<0\cdot02$
† $P<0\cdot01$, § $P<0\cdot001$

Two ovaries could not be classified. They contained loose stroma with enlarged lymph and blood vessels. A few small, non-growing and preantral follicles were seen. Antral follicles were absent. Several cysts lined with a few layers of elongated cells were present.

*The effect of the duration of the disease*

The duration of the disease (survival time) was considered in the different cases, to determine whether it has an influence on the stage of ovarian development.

Actively growing ovaries were found among children with short as well as with long survival times. However, actively growing ovaries with a normal number of large antral follicles were obtained only from children who died within a week after diagnosis (Table II). The largest follicle diameter was slightly though not significantly smaller in these ovaries. In children with survival times of 2–4 months, the number of large antral follicles and the diameter of individual follicles was reduced by about 50% compared to the controls. A longer duration of the disease did not cause a further reduction in the number or the size of large follicles. Four out of 7 of the quiescent ovaries occurred in children with a 2–4 months survival time. Three quiescent ovaries (2 of them with a reduced number of small, non-growing oocytes) and the cases with abnormal stroma and cysts, came from children who died after 1–2 years of disease.

The ovaries of the 6 children who had received radiation therapy in addition to cytotoxic drugs could not be distinguished from those treated with cytotoxic drugs only.

### DISCUSSION

All ovaries obtained from normal children showed follicle growth; none of them was quiescent. This confirms previous observations that follicle growth and atresia occur throughout infancy and childhood (Block, 1952; Valdes-Dapena, 1967; Lintern-Moore *et al.*, 1974; Peters, *et al.*, 1975, 1976;

The present findings show that most of the ovaries of leukaemic children were abnormal. Leukaemic infiltration of the ovary occurred in only one case. (It has previously been reported that leukaemic infiltration in internal organs, including the gonads, is a main complication of the disease (von Somm, 1965; Simone *et al.*, 1972; Till and Hardisty, 1973).)

Twenty-two per cent of the ovaries showed no follicle growth at the time of death (quiescent ovaries). However, the presence of "scars" of large follicles, which are the last stage of the atretic process (Watzka, 1957; Himelstein-Braw *et al.*, 1976) suggests that follicle growth and atresia had occurred previously. Actively growing ovaries among the leukaemic children were common, but they were not normal. The number and size of large antral follicles were significantly reduced in all cases (Table II). Whether follicle growth was inhibited by the disease itself or by the treatment is uncertain. However, the fact that the ovaries of the children who had been under treatment for only 1 week were normal, suggests that it is not the disease but the treatment that influenced follicle development. All ovaries of children treated for more than 2 months showed inhibition of follicle growth. Prolonged treatment (1–2 years) caused a reduction in the number of small oocytes (2 cases) and affected ovarian stroma (2 cases).

Prolonged combined treatment with corticosteroids and cytotoxic agents is known to cause damage to several internal organs and growth retardation,(von Somm, 1965; Simone *et al.*, 1972; Thunold and Moe, 1973). Alkylating agents such as cyclophosphamide have been reported to inhibit the growth of follicles, to destroy small oocytes and to induce gonadal atrophy and amenorrhoea (Sieber and Adamson, 1975; Sobrinho *et al.*, 1971; Warne *et al.*, 1973; Miller *et al.*, 1971; Miller & Cole, 1970). Cytotoxic drugs inhibit cell proliferation and growth in actively growing cells through action on different phases of the cell cycle (Spiers, 1974). The disturbance in follicle development seen in leukaemic children is probably due to a direct effect of the cytotoxic drugs on the granulosa cells of developing follicles, resulting in a retardation and inhibition of follicle growth.

This study was carried out in partial fulfilment of EURATOM contract 120–73–1 BIO DK.

### REFERENCES

Block, E. (1952) Quantitative morphological investigations of the follicular system in women. *Acta Anat.* **14**, 108.

Himelstein-Braw, R., Byskov, A. G., Peters, H. & Faber, M. (1976) Follicular atresia in the infant human ovary. *J. Reprod. Fert.*, **46**, 55.

Lintern-Moore, S., Peters, H., Moore, G. P. M. & Faber, M. (1974) Follicular development in the infant human ovary. *J. Reprod. Fert.*, **39**, 53.

Miller, J. J., III & Cole, L. J. (1970) Changes in mouse ovaries after prolonged treatment with cyclophosphamide. *Proc. Soc. Exp. Biol. Med.*, **133**, 190.

Miller, J. J., III, Williams, G. F. & Leissring, J. C. (1971) Multiple late complications of therapy with cyclophosphamide, including ovarian destruction. *Am. J. Med.*, **50**, 530.

Peters, H., Byskov, A. G., Himelstein-Braw, R. & Faber, M. (1975) Follicular growth: the basic event in the mouse and human ovary. *J. Reprod. Fert.*, **45**, 559.

Peters, H., Himelstein-Braw, R. & Faber, M. (1976) The normal development of the ovary in childhood. *Acta Endocrinol. (Kbh.)*, **82**, 617.

Sieber, S. M. & Adamson, R. H. (1975) Toxicity of antineoplastic agents in man: chromosomal aberration, antifertility effects, congenital malformations, and carcinogenic potential. *Adv. Cancer Res.*, **22**, 57.

Simone, J. V., Holland, E. & Johnson, W. (1972)

Fatalities during remission of childhood leukemia. *Blood.*, **39**, 759.

SOBRINHO, L. G., LEVINE, R. A. & DECONTI, R. C. (1971) Amenorrhea in patients with Hodgkin's disease treated with antineoplastic agents. *Am. J. Obstet. Gynec.*, **109**, 135.

VON SOMM, P. (1965) Komplikationen der akuten Leukämie im Kindesalter unter kombinierter Steroid-Cytostatica-Therapie. *Helv. Ped. Acta*, **20**, 75.

SPIERS, A. S. D. (1974) Mode of action and clinical uses of therapeutic agents in leukemia. In *Leukemia.* Eds. F. Gunz & A. G. Baike Grune & Stratton. New York: p. 561.

VON STIEVE, H. (1949) Anatomisch nachweisbare Vorgänge im Eierstock des Menschen und ihre umweltbedingte Steuerung. *Geburtshilfe Frauenheilkd.*, **9**, 639.

THUNOLD, S. & MOE, P. J. (1973) Complications of cytostatic therapy in childhood leukemia. *Acta Pathol. Microbiol. Scand.*, (A), **236**, 84.

TILL, M. M. & HARDISTY, R. M. (1973) Long survivals in acute leukaemia. *Lancet*, i, 534.

VAN WAGENEN, G. & SIMPSON, M. E. (1973) Postnatal development of the ovary in *Homo sapiens* and *Macaca mulatta* and induction of ovulation in the Macaque. New Haven, London: Yale University Press.

VALDES-DAPENA, M. A. (1967) The normal ovary of childhood. *Ann. N.Y. Acad. Sci.*, **142**, 597.

WARNE, G. L., FAIRLEY, K. F., HOBBS, J. B. & MARTIN, F. I. R. (1973) Cyclophosphamide-induced ovarian failure. *N. Engl. J. Med.*, **289**, 1159.

WATZKA, M. (1957) Weibliche Genitalorgane. Das Ovarium. Eds. W. V. Möllendorf & W. Bargmann In. *Handbuch der mikroskopischen Anatomie des Menschen. Vol. 7.* Berlin: Springer Verlag. p. 90.